# Supplementary material for: Two new species of Erythroneurini (Hemiptera, Cicadellidae, Typhlocybinae) from southern China based on morphology and complete mitogenomes
Source: PeerJ. 2024 Feb 8;12:e16853. doi: 10.7717/peerj.16853 (PMC10859084; doi:10.7717/peerj.16853)

FigS1 Inferred secondary structures of 22 tRNA from four species. Watson – Crick base pairings are illustrated by lines (-), whereas GU base pairings are illustrated by★.


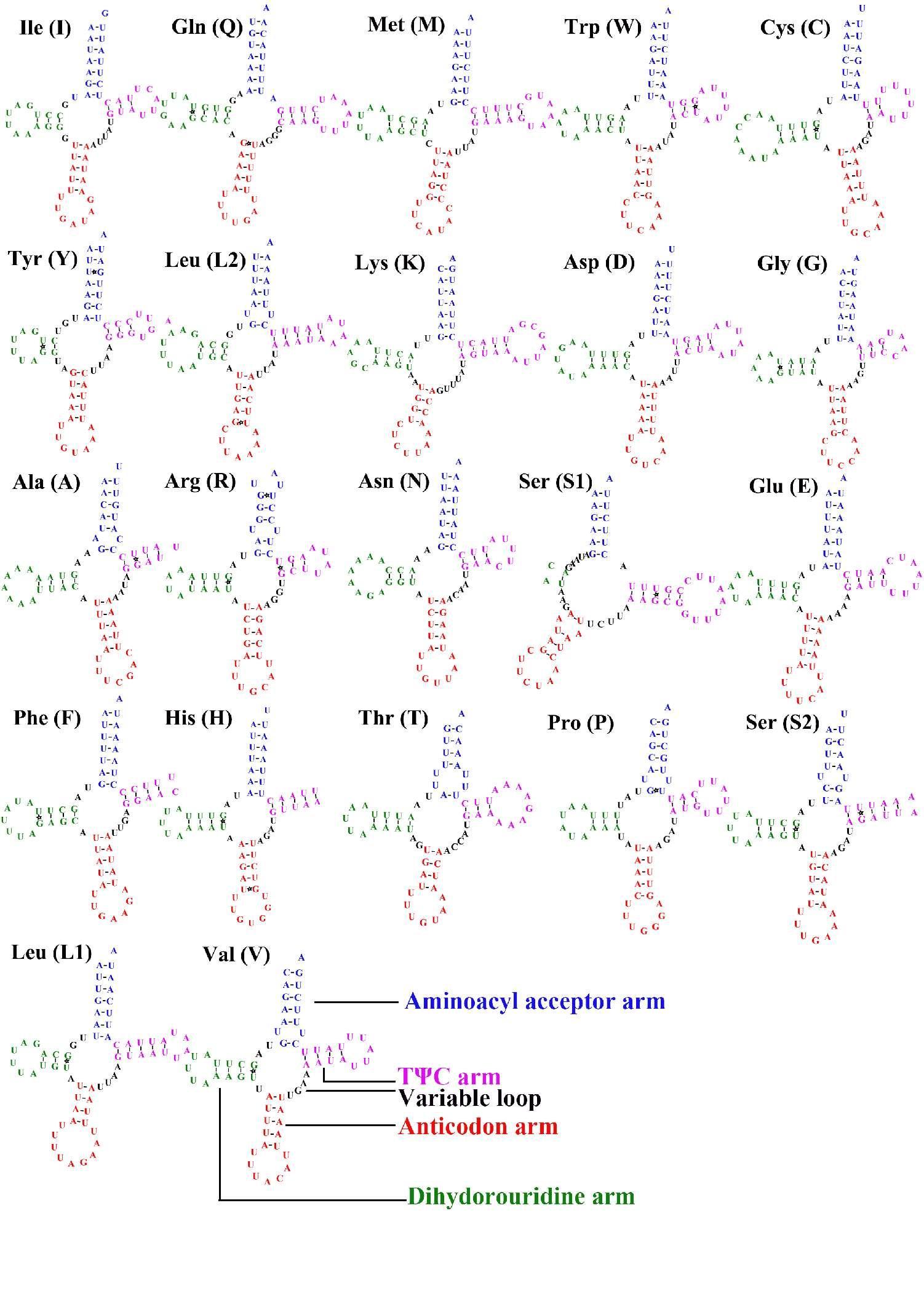


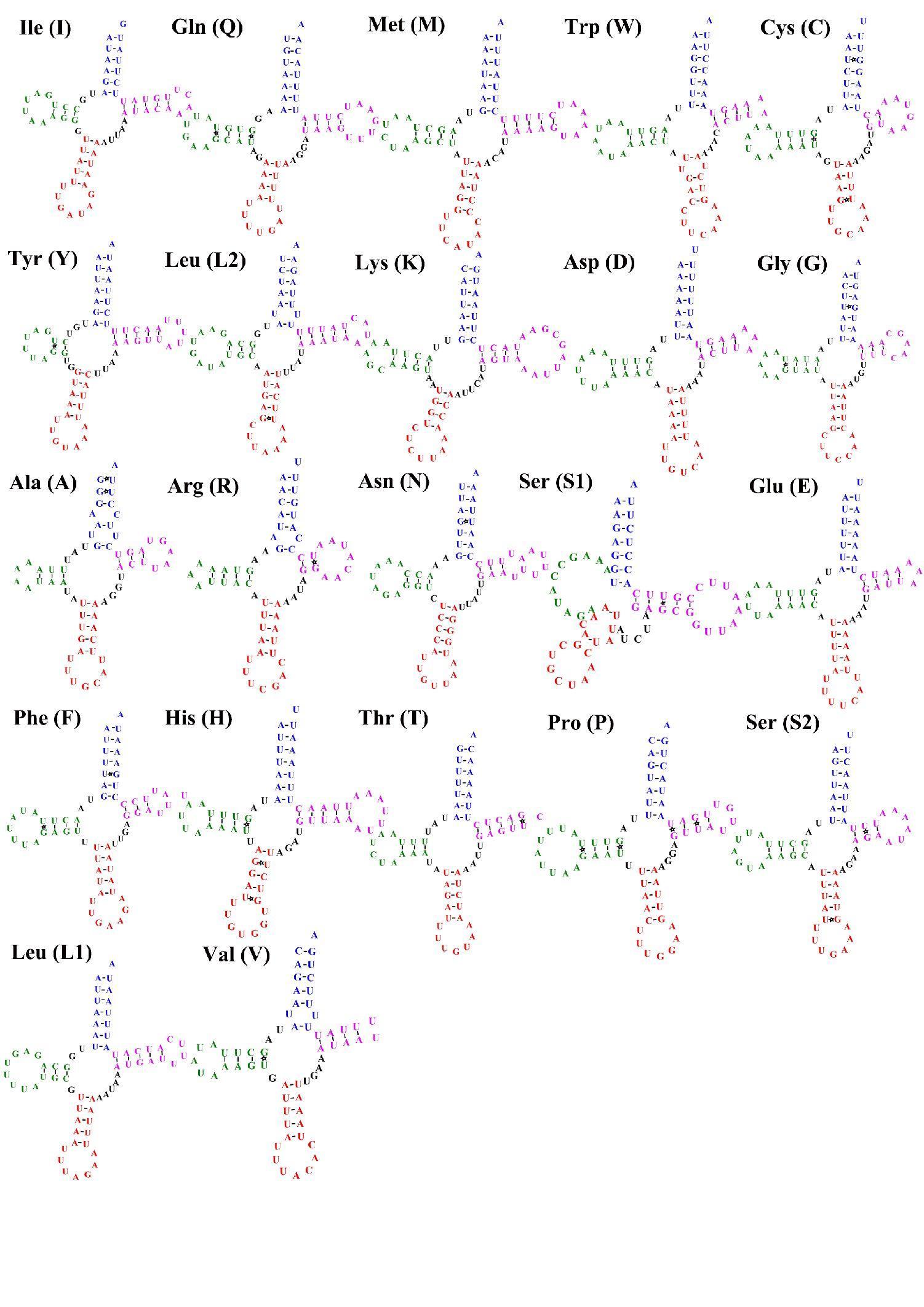

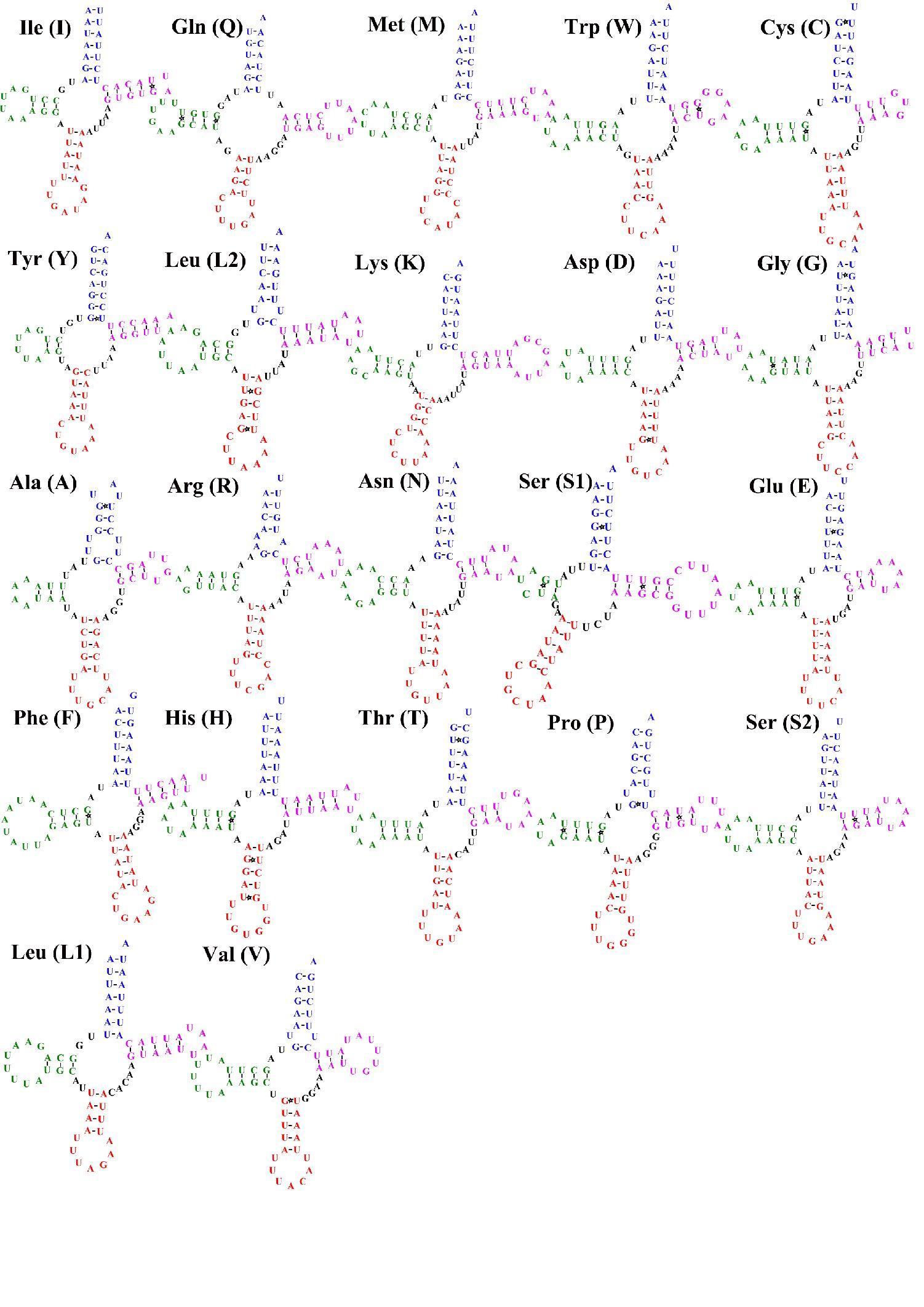

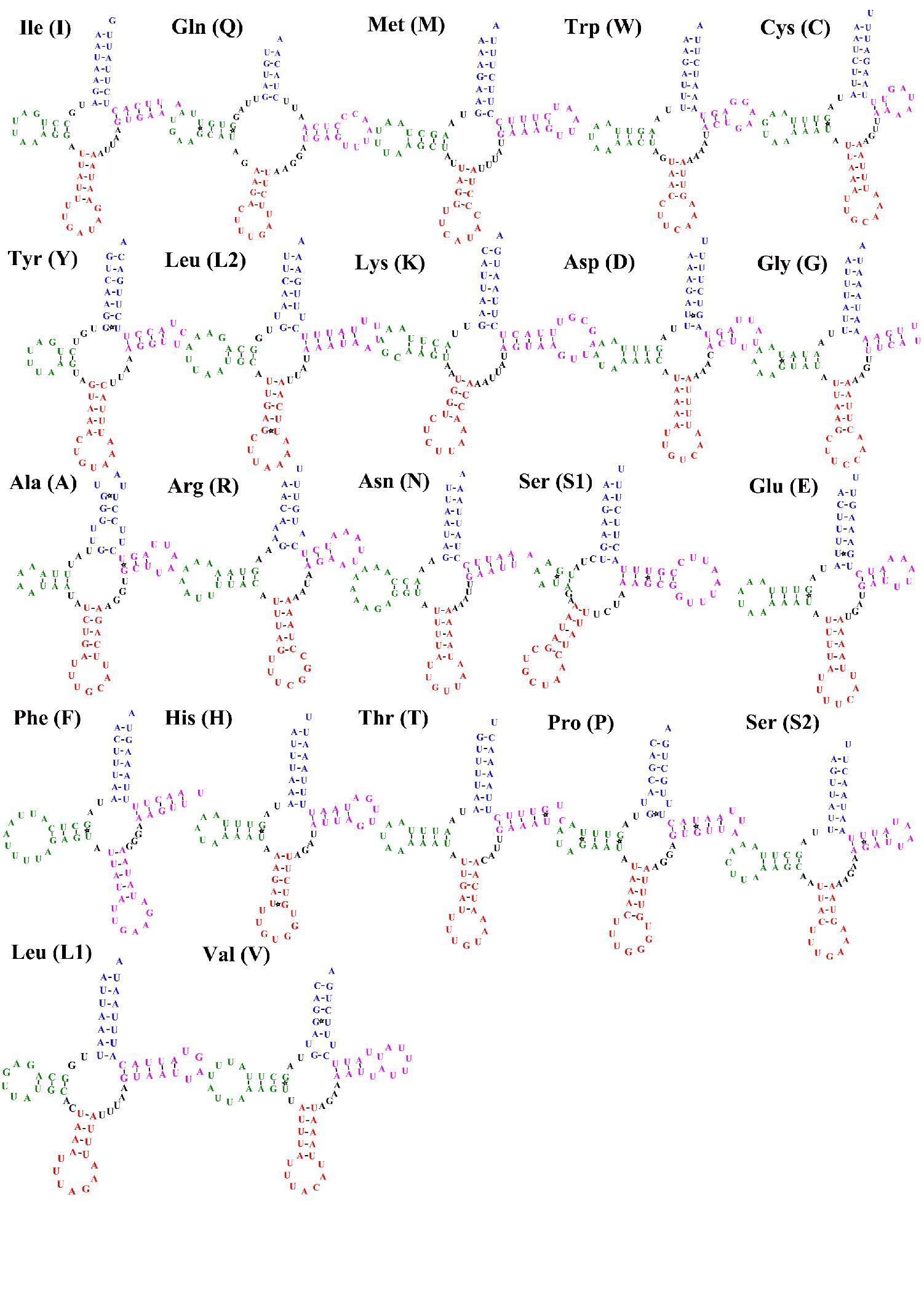

Supplement: Figure S1 — Watson–Crick base pairings are illustrated by lines (-), whereas GU base pairings are illustrated by ⋆. [file peerj-12-16853-s003.doc]
